# Selective Effect of DNA N6-Methyladenosine Modification on Transcriptional Genetic Variations in East Asian Samples

**DOI:** 10.3390/ijms251910400

**Published:** 2024-09-27

**Authors:** Meiwei Luan, Kaining Chen, Wenwen Zhao, Minqiang Tang, Lingxia Wang, Shoubai Liu, Linan Zhu, Shangqian Xie

**Affiliations:** 1 School of Basic Medicine, Harbin Medical University, Harbin 150081, China; meiweiluan@163.com; 2 Guangzhou Women and Children’s Medical Center, Guangzhou Medical University, Guangzhou 511436, China; chenkn_bioinfo@163.com; 3 College of Forestry, Hainan University, Haikou 570228, China; zhaowenwen369@126.com (W.Z.); tangminqiang@hainanu.edu.cn (M.T.); wlxhnu2000@163.com (L.W.); hainanliushoubai@163.com (S.L.); 4 School of Mechanical and Materials Engineering, Washington State University, Pullman, WA 99163, USA; linan.zhu@wsu.edu

**Keywords:** 6mA modification, DNA and RNA variation, transcriptional genetic variations, selective effect, East Asian

## Abstract

Genetic variations and DNA modification are two common dominant factors ubiquitous across the entire human genome and induce human disease, especially through static genetic variations in DNA or RNA that cause human genetic diseases. DNA N6-methyladenosine (6mA) methylation, as a new epigenetic modification mark, has been widely studied for regulatory biological processes in humans. However, the effect of DNA modification on dynamic transcriptional genetic variations from DNA to RNA has rarely been reported. Here, we identified DNA, RNA and transcriptional genetic variations from Illumina short-read sequencing data in East Asian samples (HX1 and AK1) and detected global DNA 6mA modification using single-molecule, real-time sequencing (SMRT) data. We decoded the effects of DNA 6mA modification on transcriptional genetic variations in East Asian samples and the results were extensively verified in the HeLa cell line. DNA 6mA modification had a stabilized distribution in the East Asian samples and the methylated genes were less likely to mutate than the non-methylated genes. For methylated genes, the 6mA density was positively correlated with the number of variations. DNA 6mA modification had a selective effect on transcriptional genetic variations from DNA to RNA, in which the dynamic transcriptional variations of heterozygous (0/1 to 0/1) and homozygous (1/1 to 1/1) were significantly affected by 6mA modification. The effect of DNA methylation on transcriptional genetic variations provides new insights into the influencing factors of DNA to RNA transcriptional regulation in the central doctrine of molecular biology.

## 1. Introduction

DNA genetic variations are ubiquitous across the genomes of all organisms and involve population stratification [1,2,3], species evolution [4,5] and human diseases [6,7]. Hundreds of thousands to millions of DNA variants in population genetics have been modeled to identify the genetic mechanisms between genotypes and phenotypes in genome-wide association studies, which have revolutionized the field of human biology and disease [8,9,10], plant environmental adaptation and so on [11,12]. For the correlation between DNA variants and phenotypes, transcriptome-wide association research provides another solution that examines RNA expression as a phenotype at the transcriptome level [13]. RNA genomic features include gene expression and RNA variants [14,15,16]. RNA variants have been widely investigated in biological transcriptome events, including germlines, stem cells, human diseases and cancers [15,17,18]. For example, the G > A transition variant in position-1 of intron 7 was used as an alternate acceptor splice site and led to the retention of intron 7 in the transcript of the keratin 5 (*KRT5*) gene, which directly downregulated *KRT5* expression in a heritable skin disorder [18]. DNA and RNA variants are important genetic variations associated with phenotypes and have been widely studied [19]. However, the relationship between DNA and RNA variants and the dynamic transcriptional genetic variations from DNA to RNA during the transcription process, requires further study.

Transcriptional initiation and elongation replicate RNA from alternative double strands of homologous chromosome DNA in diploid organisms [20]. Transcription begins at well-defined transcription start sites and proceeds in one direction to synthesize RNA. Transcription start sites and RNA variants are two influencing transcription factors [21,22]. RNA variants affect transcription elongation. For example, the expansion of (GAA)n repeats within the first intron of the frataxin (*FTX*) gene block transcription elongation due to the tardiness of the RNA polymerase in the variation region [21]. Most RNA variants come from DNA; therefore, the transcriptional genetic variations from DNA to RNA directly regulate transcription and gene expression, especially for heterozygous genetic variation [23,24,25,26,27]. The accumulation of variants in transcription is enabled by the asymmetry of heterozygous variations that produce compositional asymmetry on the coding strand [28,29,30]. Genetic variants associated with differences in mRNA levels are affected by epigenetic and transcriptional regulation [31]. An analysis of transcriptomes from 982 acute myeloid leukemia patients showed that RNA variants in isocitrate dehydrogenase (NADP(+)) 2 (*IDH2*) and serine- and arginine-rich splicing factor 2 (*SRSF2*) promoted leukemogenesis and epigenome and RNA splicing [32]. Smoking-induced epigenetic changes in G protein-coupled receptor 15 (*GPR15*) are correlated to the gene expression profile [33]. Several studies have reported the interaction influence of gene expression, DNA methylation and genetic variations [34,35,36,37]. Methylation expression patterns have been established from DNA methylation and transcriptome sequencing data in GenCord human populations [35]. Association analyses of methylation levels with variations have shown that methylation affects gene expression in HapMap cell lines [36]. Epigenetic effects and imprinting have been studies from placental samples helps to explain the random monoallelic effects in the expression patterns [38]. At present, the existing research results directly confirm that DNA methylation affects transcription.

DNA methylation refers to the modification of a methyl group to all four types of DNA nucleotides. The carbon-5 position of cytosine (5-methylcytosine, 5mC) and nitrogen-6 position of adenine (N6-methyladenosine, 6mA) are the most abundant DNA modifications in eukaryotes and prokaryotes [39,40,41]. 5mC has been widely studied in the genomes of most eukaryotes and is an important epigenetic mark relevant to gene regulatory systems [42,43]. An examination of 5mC methylation levels with common single-nucleotide polymorphisms identified 180 CpG sites in 173 genes that were associated with nearby single-nucleotide polymorphisms (putatively in *cis*, usually within 5 kb) [36]. 5mC is involved in genetic variation and is directly connected with genetically regulated gene expression in the human brain [44]. Recently, several studies have revealed diverse functions affected by DNA 6mA modifications in eukaryotes, including the regulation of gene expression [45,46,47,48,49,50], transposons expression [49,51,52] and cross-talk with histone modifications [53,54]. The existence of 6mA modifications as a new epigenetic mark creates a novel paradigm in epigenetics and epigenomics regarding the regulation of biological processes in the eukaryotic systems [49,55]. 6mA-IP-qPCR and 6mA-RE-qPCR occur on 6mA and liquid chromatography–tandem mass spectrometry (LC-MS/MS) has been used to measure the 6mA ratio in human cells, which was developed for 6mA identification [50]. The DNA methyltransferase *N6AMT1* and demethylase *ALKBH1* mediate the methylation and demethylation of DNA 6mA in the human genome, which are the structural basis of nucleic acid recognition and 6mA demethylation [56]. 6mA cross-linking exonuclease sequencing (6mACE-seq) utilizes 6mA-specific antibodies cross-linked to 6mA sites and allows the mapping of human-genome-wide 6mA at a single-nucleotide resolution [57]. The novel 6mA modification has been identified in a highly malignant brain cancer glioblastoma [54]. SMRT DNA sequencing is popular for directly identifying 6mA modifications based on the inter-pulse duration (IPD) during DNA synthesis [55,58], which has advantages in identifying 6mA sites at a strand-specific and single-nucleotide resolution [46,48,53]. DNA 6mA modification plays a regulatory role in gene expression [40,50,59] and is closely related to embryonic development [52,60]. In human studies, the global 6mA DNA level in leukocytes was significantly decreased in hypertension patients and increased in esophageal squamous cell carcinoma [61,62]. DNA 6mA modification modulated stress responses and signal transgenerational inheritance in *Caenorhabditis elegans* [63]. Genome-wide 6mA and transcriptome profiling revealed an inverse association between 6mA dynamic changes and a set of upregulated neuronal genes or downregulated LINE transposon expression in neuropsychiatric disorders [51]. For the role of DNA 6mA methylation in the transcriptional variants, our previous study revealed that RNA variants preferred homozygous when transcribed from heterozygous DNA on the genes with DNA 6mA methylation in the plant *Herrania umbratica* [37]. However, the effects of DNA 6mA modification on dynamic transcriptional genetic variations remain largely undiscovered in humans.

In this study, we identified DNA, RNA and transcriptional genetic variations from Illumina short-read sequencing data in East Asian samples (HX1 and AK1) and detected global DNA 6mA modification using SMRT sequencing data. We decoded the effects of DNA 6mA modification in transcriptional genetic variations of the East Asian samples (HX1 and AK1) and extensively verified the results in the HeLa cell line. DNA 6mA methylation had a stabilized distribution in the East Asian samples and a selective effect on genetic variation transmission from DNA to RNA during transcription.

## 2. Results

### 2.1. Identification of DNA 6mA Modification in East Asian Samples

**Figure 1:**
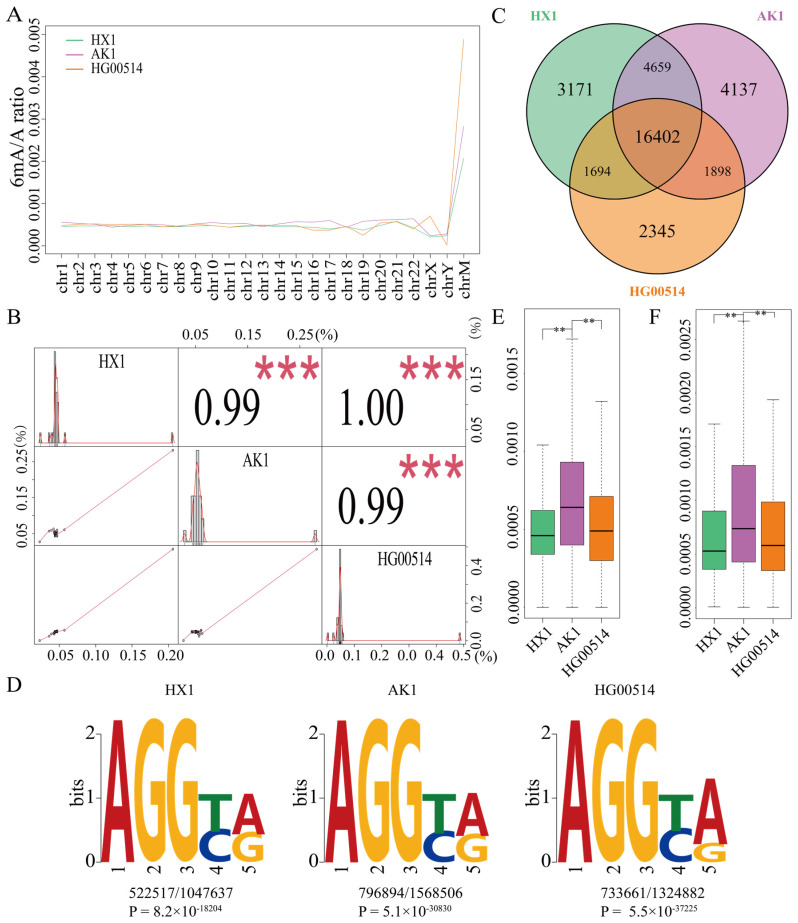
DNA 6mA modification in HX1, AK1 and HG00514 genomes ((**A**) 6mA density modification in each chromosome; (**B**) Correlation coefficient of any two samples and distribution of 6mA density in chromosomes; (**C**) Venn diagram of the 6mA methylated genes in three samples; (**D**) Motif of 6mA modification sites in the consistent genes in three samples; (**E**) Boxplot of 6mA density in the consistent methylated genes, (**F**) Boxplot of 6mA density in methylated genes, ** refer to *p*-value < 0.01, *** refer to *p*-value < 0.001).

The 6mA density of the entire genome in the three samples HX1, AK1 and HG00514 was 0.043%, 0.049% and 0.047%, respectively (Tables S1 and S2) and the densities of each chromosome were almost consistent (Figure 1A and Table S2). The correlation coefficient of any two samples was more than 0.99 among the three samples (Figure 1B). Therefore, DNA 6mA modifications were almost consistent in the East Asian samples. The correlation of the two Chinese samples (HX1 and HG00514) was particularly consistent (1.00) compared to the correlation between the Chinese and Korean samples. The total numbers of methylated genes with 6mA modification (same strand) were 25,926, 27,096 and 22,339 in HX1, AK1 and HG00514, respectively (Figure 1C and Table 1). Among them, 16,402 methylated genes were consistent in three samples and these shared genes accounted for 63.26%, 60.53% and 73.42% in HX1, AK1 and HG00514, respectively. Comparing the methylated genes between the Chinese and Korean samples, we identified 21,061 shared genes in HX1 and AK1 that accounted for 81.24% of HX1 methylated genes. A total of 18,300 shared methylated genes of HG00514 and AK1 accounted for 81.92% of methylated genes in HG00514 (Figure 1C). To further validate the consistency of the 6mA modification, we compared and statistically analyzed the 6mA density (6mA/A) of methylated genes and consistent methylated genes among the three samples using Student’s *t*-test. The 6mA density of AK1 was significantly higher than that of HX1 and HG00514 (Figure 1E,F, *p*-value < 2.2 × 10^−16^). The 6mA density of whole methylated genes between the Chinese samples (HX1 and HG00514) was not significant (Figure 1E) and the density of the consistent methylated genes (16,402) was significantly different (Figure 1F, *p*-value = 1.083 × 10^−4^). These results confirmed the rationality and reliability of methylation identification in the East Asian samples.

Conserved DNA sequence motifs of short nucleotides are widespread around the 6mA modification sites in the human genome [50]. To confirm the motif pattern among HX1, AK1 and HG00514, the significant enrichment of consensus motifs was searched using MEME [64]. The prominent motif sequences of the 6mA sites were detected in the consistent methylated genes. The AGGYR motif was significantly enriched in all three samples (Figure 1D), consistent with the previous motif sequence reported in humans [50].

### 2.2. Effect of 6mA Modification on DNA Variations

**Figure 2:**
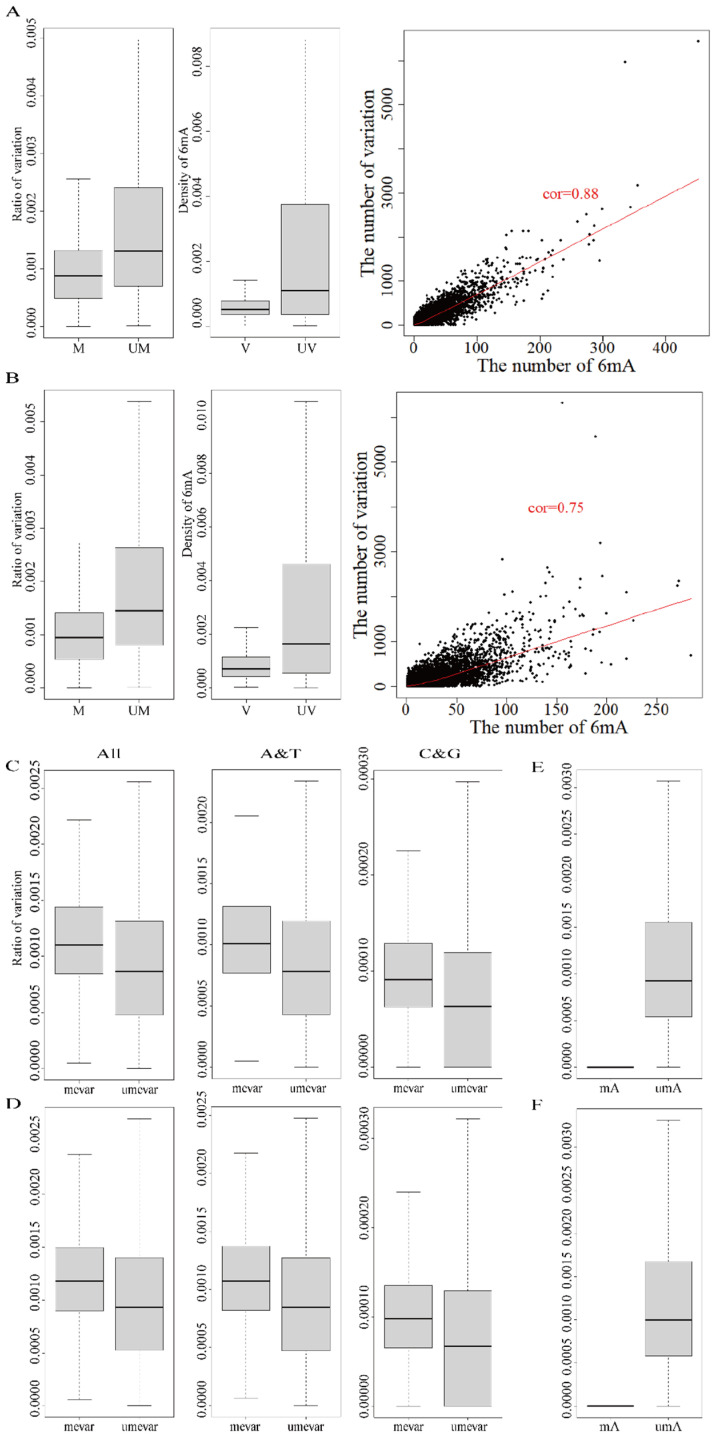
Effect of 6mA modification in DNA variations in HX1 and AK1. ((**A**) Correlation of 6mA modification density and ratio of variations in HX1; (**B**) Correlation of 6mA modification density and ratio of variations in AK1; (**C**) Comparison of the variation ratios in the methylated and unmethylated variation genes in HX1; (**D**) Comparison of the variation ratios in the methylated and unmethylated variation genes in AK1; (**E**) Comparison of A-mutated ratios (mutated-A/A) in A sites with modified and unmodified 6mA in same genes of HX1; (**F**) Comparison of A-mutated ratios (mutated-A/A) in A sites with modified and unmodified 6mA in same genes of AK1. cor: refers to correlation, UM: unmethylated genes, M: methylated genes, V: variation genes, UV: non-variation genes, mevar: methylated variation genes, umevar: unmethylated variation genes, mA: A-mutated with methylated, uMA: A-mutated with unmethylated).

The East Asian samples (HX1 and AK1) were selected to subsequently analyze the relationship between DNA methylation and genetic variations. We identified and validated 2,593,902 and 2,788,637 DNA variations in HX1 and AK1, respectively (Table 2 and Table S3). Among these variations, 1,522,416 and 1,638,029 variants occurred in 35,232 and 35,685 genes of HX1 and AK1, respectively (Table 2 and Table S3). The DNA variation ratio of each gene was calculated by the number of variations in the length of the corresponding gene. The results showed that the mean ratio of DNA variations in the unmethylated genes (genes without 6mA modification) and methylated genes were 0.21% and 0.10% in HX1, respectively (Table S3). Similarly, the mean ratios of DNA variations of unmethylated genes and methylated genes were 0.22% and 0.11% in AK1, respectively. The slight difference (0.01%) of variation between HX1 and AK1 was normal and similar to the previous 6mA modification. The comparison of DNA variations between unmethylated and methylated genes showed that the variations in the unmethylated genes were significantly higher than in the methylated genes (Figure 2A,B and Table S4, *p*-value < 2.2 × 10^−16^). Furthermore, the 6mA density of genes with DNA variations was lower than that in non-variation genes (Figure 2A,B and Table S3, *p*-value < 2.2 × 10^−16^). The results indicated that the DNA 6mA modifications lowered the gene mutation and promoted gene stability. This effect on 6mA modification was consistent with previous studies in *H. umbratica* [37].

The consistent methylated and mutated genes in HX1 (22,280) and AK1 (23,293) were subsequently analyzed. The number of 6mA modification loci and variations had a significant positive correlation (*p*-value < 2.2 × 10^−16^) and Pearson’s correlation coefficient was 0.88 for HX1 and 0.75 for AK1 (Figure 2A,B and Table 2). We detected 2270 and 2308 DNA variations that were also methylation sites (methylated variation) in genes from HX1 and AK1, respectively (Table 2, Tables S4 and S5). We compared the variation ratio of genes between the methylated variation genes (methylated and mutated genes with 6mA modification A base variation, 707 in HX1 and 809 in AK1) and the unmethylated variation genes (methylated and mutated genes without 6mA modification, 21,573 in HX1 and 22,484 in AK1) (Table 2). The mean ratio of variations in the methylated variation genes was 0.12% and 0.13% in HX1 and AK1, which was significantly higher than that in unmethylated variation genes (0.10% and 0.11%) (*p*-value < 2.2 × 10^−16^, Figure 2C,D and Tables S3–S5). 6mA modification occurs at the A site; therefore, we selected A and T variations to subsequently study the effect of 6mA modification for the variation at the A and T sites. The mean ratio of A and T variation in the methylated variation genes was 0.11% in HX1 and AK1, which was higher than that in the unmethylated variation genes (0.07%) (*p*-value < 2.2 × 10^−16^, Figure 2C,D and Tables S3–S5). Similar results have appeared on C and G variation in methylated and unmethylated variation genes (*p*-value < 2.2 × 10^−16^, Figure 2C,D and Tables S3–S5). The comparison of the A-mutated ratio between modified and unmodified 6mA showed the mutated ratio of 6mA was significantly less than the unmodified A base mutation (Figure 2E,F). Therefore, the effect of 6mA modification was significantly positively associated with DNA genetic variations and the effect was cis-regulation and resistant mutation.

### 2.3. Effect of 6mA Modification on RNA Variations

We identified 21,346 and 80,139 RNA variations in HX1 and AK1, respectively (Table 3 and Table S3). Among these RNA variations, 21,044 in HX1 and 77,791 in AK1 occurred on 6630 and 13,129 genes, respectively (Table 3). Therefore, the mean ratios of RNA variations in the unmethylated and methylated genes were 0.242% and 0.028% in HX1, respectively (Table S3). Similarly, the mean ratios of RNA variations in unmethylated and methylated genes were 0.387% and 0.051% in AK1, respectively (Table S3). The ratio of variations in the unmethylated genes was almost six times higher than the ratio in the methylated genes in HX1 and AK1; therefore, DNA 6mA modification had strong effects on RNA variation (Figure S1). The results showed that the ratio of RNA variation in the methylated genes was significantly lower than that in the unmethylated genes (*p*-value < 2.2 × 10^−16^, Figure S1), which was similar to that described for DNA.

### 2.4. Selective Effect of 6mA Modification on Transcriptional Variations from DNA to RNA

RNA genetic variations occur from DNA by the biological transcription process under the central principle of molecular biology [65]. Theoretically, transcriptional genetic variations of “DNA makes RNA” in the central doctrine are random and RNA variations are derived from DNA variations. In HX1 and AK1 samples, we detected 21,346 and 80,139 RNA variations, respectively (Table 3 and Table S3). Less than 50% of these in HX1 (7631) and AK1 (28,787) were transcriptional genetic variations from DNA to RNA (Table 3). The effect of 6mA modification on static DNA and RNA variations was not similar, indicating that DNA methylation might have an important effect on transcriptional genetic variations from DNA to RNA. Our previous study showed that DNA 6mA methylation effectively influenced the transmission of transcriptional genetic variation in *H. umbratica*. To further investigate the effect of 6mA on transcriptional variations in humans, we analyzed the transcriptional variations from DNA to RNA, including variations from DNA homozygous to RNA homozygous (1/1-0/0 and 1/1-1/1), homozygous to heterozygous (1/1-0/1), heterozygous to homozygous (0/1-0/0 and 0/1-1/1) and heterozygous to heterozygous (0/1-0/1), where 0 and 1 were the normal and mutated bases, respectively.

There were 21,346 and 80,139 transcriptional genetic variations in HX1 and AK1, respectively (Table 3). To make the analysis more convincing, we selected 18,519 consistent transcriptional variations as high-confidence variations that were simultaneously detected in HX1 and AK1(Table 3) and selected 142,254 consistent 6mA sites as high-confidence methylation sites from HX1 and AK1. Then, we calculated the 6mA density of related genes, including 142,254 consistent 6mA sites and six type numbers of transcriptional variations.

A logistic regression model was applied to classify the 6mA density of the genes (see Section 4). The logistic regression model results showed that the methylated gene was associated with the transcriptional variation types 0/1 to 0/1 and 1/1 to 1/1 (*p*-value < 0.001, Table 4, Tables S6 and S7). This effect of DNA 6mA modification demonstrated that 6mA maintained the stability of variation transmission in HX1 and AK1.

For methylated genes, we then evaluated the effect of 6mA density on the number of transcriptional variations in the genes. A linear regression model of 6mA density and number of variations showed that the methylated genes were associated with the transmitted variation types 0/1 to 0/0 (*p*-value < 0.0001, Table 5, Tables S6 and S7). Therefore, the transcriptional variations were regulated and specifically selected from DNA to RNA by DNA 6mA modification.

To further verify the selective effect of 6mA methylation in transcriptional genetic variations, we singled out the immortal cell line, HeLa, which is derived from cervical cancer cells in African American women and widely used in the study of human genetics. First, we screened out 11,352 consistent transcriptional genetic variations from HX1 and AK1 (Tables S8 and S9). A logistic regression model was applied to classify methylated genes, showing they were associated with the transcriptional variation types 0/1 to 0/1 in HeLa cells (Table 6). These results from the HeLa cell line were consistent with the above conclusion that 6mA modification stabilized genes and had a selective effect on transcriptional genetic variations in East Asian and African American samples. The selective effect of 6mA methylation might be a universal phenomenon in human genomes. Methylation might have a selective effect on transcriptional genetic variations in the central doctrine of molecular biology: “DNA makes RNA”.

### 2.5. Validation of 6mA Methylation Effect on Variations in Imprinting Genes

For the selective effect of DNA 6mA methylation, transcriptional genetic variations showed a favorable transmission. To further validate the effect of methylation on genetic variations, we collected imprinting genes (204) and non-imprinting genes (58,473). The imprinting genes (methylated and unmethylated) and non-imprinting genes (methylated and unmethylated) were compared in DNA variation number and the results of variation count showed that the *p*-value of significant level between imprinting and non-imprinting genes in the methylated genes (HX1: 0.01, AK1: 4.28 × 10^−3^) was stronger than those between imprinting and non-imprinting genes in the unmethylated genes (HX1: 0.22, AK1: 0.04, Table 7, Tables S10 and S11). Moreover, the variation number between imprinting and non-imprinting genes in the unmethylated genes was not significant in HX1 (*p*-value = 0.22, Table 7, Tables S10 and S11). For future explore the 6mA modification effect on genetic variation, the significance of the DNA variation number between the methylated (imprinting and non-imprinting) and unmethylated genes (imprinting and non-imprinting) was compared for HX1 and AK1. The results showed that the variation number of methylated genes in HX1 and AK1 was significantly lower than unmethylated genes in both imprinting and non-imprinting genes (Table 8). In consistent transmitted variations, the comparison of imprinting and non-imprinting genes revealed no significant differences (Tables S11 and S12). These results further confirmed that DNA 6mA modification indeed influenced DNA variants and transcriptional variations, the imprinting genes possibly affected the genetic variation for methylated genes and the imprinting genes had no effect on transmitted variations.

### 2.6. Relationship between 6mA Modification and Variations in Coding and Regulated Regions

**Figure 3:**
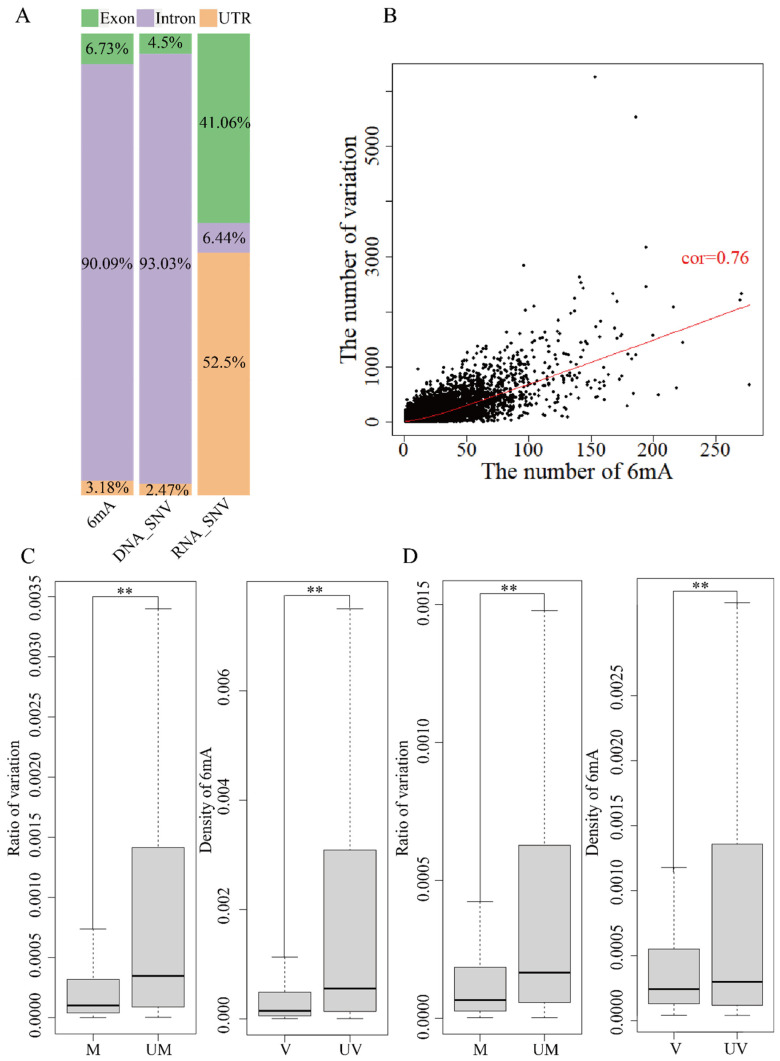
Effect of 6mA modification and variation in genome different region in HX1. (**A**) Distribution rate of 6mA and variation in exons, introns and UTRs; (**B**) Correlation of 6mA modification density and ratio of variations in intron regions; (**C**) Comparison of the DNA variation ratios in the methylated and unmethylated variation genes and 6mA density in DNA mutated and unmutated genes in exons; (**D**) Comparison of the RNA variation ratios in the methylated and unmethylated variation genes and 6mA density in RNA mutated and unmutated genes in exons, ** refer to *p*-value < 0.01, cor: refers to correlation, UM: unmethylated genes, M: methylated genes, V: variation genes, UV: non-variation genes).

**Figure 4:**
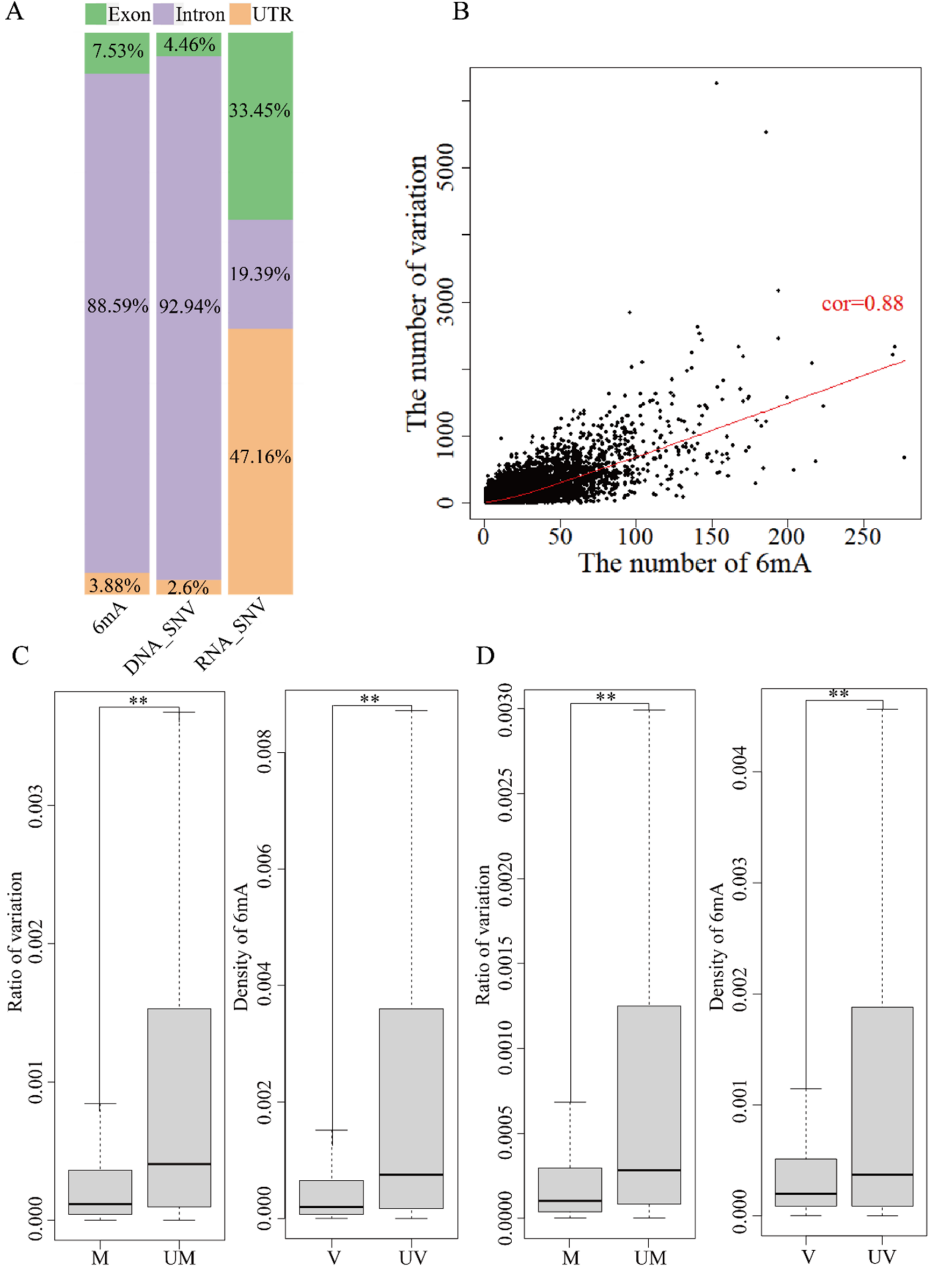
Effect of 6mA modification and variation in genome different region in AK1. ((**A**) Distribution rate of 6mA and variation in exons, introns and UTRs; (**B**) Correlation of 6mA modification density and ratio of variations in intron regions; (**C**) Comparison of the DNA variation ratios in the methylated and unmethylated variation genes and 6mA density in DNA mutated and unmutated genes in exons; (**D**) Comparison of the RNA variation ratios in the methylated and unmethylated variation genes and 6mA density in RNA mutated and unmutated genes in exons, ** refer to *p*-value < 0.01, cor: refers to correlation, UM: unmethylated genes, M: methylated genes, V: variation genes, UV: non-variation genes).

A different region of the genome contains UTR, intron and exon based on the reference genome annotation file (GRCh38.p12). 6mA modification and DNA variation were about 90% on intron regions, less than 5% on UTR and 5~7% on exons (Figure 3A and Figure 4A). About 50% of RNA variation was on exons and UTR and only 6~20% was on introns. The analysis of the relationship between 6mA modification and variations showed similar results for global genes (Figure 3B,C and Figure 4B,C). The mean ratio of DNA variations in the unmethylated genes and methylated on exon and UTR regions showed that the variations in the unmethylated genes were significantly higher than in the methylated genes (Figure 3C and Figure 4C and Table S13, *p*-value < 0.01); the mean ratio of DNA variations on intron regions had no significant differences. Furthermore, the 6mA density of exon, intron and UTR regions with DNA variations was lower than that in non-variation genes (Figure 3A,B and Figure 4A,B and Table S13, *p*-value < 2.2 × ^−16^). The results indicated that the DNA 6mA modifications lowered the exon mutation and promoted gene stability. The concurrence methylated and mutated genes in HX1 (22,280) and AK1 (23,293) were subsequently analyzed. The number of 6mA modification loci and variations on intron regions had a significant positive correlation (*p*-value < 2.2 × ^−16^) and Pearson’s correlation coefficient was 0.88 for HX1 and 0.76 for AK1, respectively (Figure 3B and Figure 4B). Exons were an important protein coding region and RNA variation of exons influenced the biological process. The mean ratios of RNA variations on exon regions were 0.14% and 0.04% in the unmethylated and methylated genes in HX1, respectively. Similarly, the mean ratios of RNA variations in the unmethylated and methylated genes were 0.21% and 0.06% in AK1, respectively. The ratio of RNA variation in the methylated genes was significantly lower than that in the unmethylated genes (*p*-value < 2.2 × 10^−16^, Figure 3D and Figure 4D) and the 6mA density of exon regions with RNA variations was lower than that in non-variation genes (Figure 3D and Figure 4D, *p*-value < 2.2 × 10^−16^), which was similar with DNA variants. It was similar to genes in that 6mA modification regulated transmitted variation from DNA to RNA on exons. A logistic regression model was applied to classify 6mA density and showed that the methylated gene was associated with the transcriptional variation types 0/1 to 0/1 and 1/1 to 1/1 (*p*-value < 0.001). This effect of DNA 6mA modification demonstrated that 6mA maintained the stability of variation transmission in HX1 and AK1.

## 3. Discussion

The central doctrine of molecular biology is an important principle of genetic information inheritance within a biological system [65]. DNA and RNA genetic variations are the main influencing factors in the transcription and genetic information inheritance [31]. DNA methylation is an important epigenetic component that is relevant to gene regulation and transcription. In the present study, we first investigated the relationship between DNA 6mA modification and genetic variations in East Asian samples, containing two aspects: (1) the association between 6mA modification and DNA/RNA variations and (2) the effect of 6mA modification in transcriptional genetic variants from DNA to RNA. DNA 6mA modification lowered the gene mutation and promoted gene stability for three samples (HX1, AK1 and HG00514) (Figure 1D). Furthermore, 6mA modification retained the transmitted mutated alleles in heterozygous (0/1 to 0/1) and homozygous (1/1 to 1/1) variations from DNA to RNA in 6mA modification genes (Table 4, Table 5 and Table 6). These findings provide new insight into the influencing factors of DNA to RNA transcriptional regulation.

DNA 6mA methylation is a newly discovered type of methylated modification and has been studied in prokaryotes and eukaryotes. Although some studies have suggested that 6mA is not present in eukaryotes [66], there is much evidence for detecting and confirming the existence of DNA 6mA modification in eukaryotes, including fungi, plants, animals and even in mammalian [40,46,49,50,52,53,54,61,67,68,69,70,71,72,73,74,75]. DNA 6mA modification is mediated by methyltransferase *N6AMT1* and demethylase *ALKBH1* and a decrease in the genomic DNA 6mA promotes tumorigenesis in human cells [50]. Using the 6mACE-seq, a human-genome-wide 6mA map reproduced known 6mA enrichment at active retrotransposons and identified a 6mA-binding protein in single-stranded DNA-binding protein 1 [73]. Chromatin immunoprecipitation with anti-6mA antibody showed that 6mA modification played an important role in the growth and development of embryos and was absent in abortuses with monosomy 21 [75]. Genomic DNA was isolated from peripheral blood mononuclear cells of 20 systemic lupus erythematosus (SLE) samples using 6mA immunoprecipitation sequencing (6mA-IP-Seq), which found that a high level of 6mA participated in the pathogenesis of SLE [72]. Several previous studies have validated that 6mA modifications are universal in human genomes and simultaneously cross-detected by multiple technologies in 6mACE-seq, 6mA-IP-Seq and SMRT sequencing. The developed SMRT sequencing technology provides a high-resolution strategy to identify the 6mA modification base using a specific IPD signal during the DNA synthesis [74]. Due to the advantage of DNA 6mA modification at the single-nucleotide resolution, the dataset used in the present study for 6mA methylation detection was obtained from SMRT sequencing (PacBio RSII). Based on previous study results and technical basis, the data and conclusions from the present study are reliable and reasonable.

Recently, the methylation effect has become a hot research topic. Several studies have found that DNA 6mA methylation occurs in genome-wide *Arabidopsis*, rice, mice and the human genome [40,46,50,59] and 6mA modification influenced adduct formation by DNA polymerases in mammalian DNA [76]. Recently, DNA 6mA modifications have been shown to lower gene mutation and promote gene stability for the selective effect of DNA 6mA modification on transcriptional genetic variations in plant *H. umbratical* [37]. In the present study, we further confirmed the selective effect of methylation at the individual level on the human genome. This research greatly expands the depth and breadth of methylation studies and provides new insight into the combination of different multiple omics data with methylated modification in humans.

Due to individual personalization, the special genes of three samples were 2944, 3847 and 2338 in HX1, AK1 and HG00514, respectively (Figure 1C). Annotating these genes using DAVID found that the results were consistent with the 6mA density modification, in which HX1 and HG00514 have similar functions on olfactory receptor activity and G-protein coupled receptor but are different from AK1 on translation and ribosome processes (Table S14). We analyzed the intersection of each of the two samples, e.g., HX1 and HG00514, HX1 and AK1 and HG00514 and AK1 and found that the function of intersected genes in HX1 and HG00514 was significantly enriched in olfactory receptor activity and the G-protein coupled receptor. However, this function was not significantly enriched in the other two sample intersections of AK1. They were consistent in 6mA density and the AK1 (Korean sample) was different from the Chinese Han samples (HX1 and HG00514). These differences between the Korean and Chinese samples should be further studied in population genetics in the future.

Imprinting genes are a small subset of genes, in which one copy is turned off in a parent-of-origin-dependent manner and refers to the epigenetic marking of the genes. Imprinting genes are an essential key to epigenetic research and help to understand the biological implications of epigenetics better. The most common molecular event in Beckwith–Wiedemann syndrome (BWS) is the biallelic expression of insulin-like growth factor 2 (*IGF2*) due to loss of imprinting (LOI), which is accompanied by methylation and/or silencing of the active maternal allele of H19 [77,78,79,80]. In a small percentage of Prader–Willi syndrome (PWS) patients who retain both parental copies of 15q11-q13, imprinting defects and promoter methylation have been identified, resulting from microdeletions targeted at the small nuclear ribonucleoprotein polypeptide N (*SNRPN*) gene [81,82,83]. DNA variation number of imprinting genes was significantly lower than non-imprinting genes in the methylated genes. The imprinting genes possibly affected the genetic variation for methylated genes. The molecular basis of epigenetic therapy for imprinting disorders involves reactivating disease-causing genes from the silenced allele at imprinted loci through pharmacological or genetic manipulation [84,85,86].

## 4. Materials and Methods

### 4.1. DNA Methylation Samples

Three East Asian samples (HX1, AK1 and HG00514) were collected from the NCBI SRA database (Table S1). HX1 and HG00514 were Han Chinese samples and AK1 was a Korean sample. The long-read sequencing data of HX1, AK1 and HG00514 from the PacBio platform were obtained from the SRA database through the bioproject numbers PRJNA301527, PRJNA298944 and PRJEB12236, respectively (Table S1) [87,88,89].

### 4.2. Identification of 6mA Modifications in Genomic DNA

For PacBio data of HX1, AK1 and HG00514, the PacBio SMRT (version 2.3.0) analysis platform was used to detect DNA 6mA methylation (https://www.pacb.com/products-and-services/analytical-software/epigenetics; accessed on 3 September 2018). Briefly, raw data of long-read DNA were aligned to the GRCh38 reference genome by pbalign with the parameters ‘−seed=1 −minAccuracy=0.75 −minLength=50 −concordant −algorithmOptions=”−useQuality” −algorithmOptions=′ −minMatch 12 −bestn 10 −minPctIdentity 70.0′’. Then, loadChemistry.py and loadPulses scripts were used to load the reagent and electrical signal of sequencing with ‘-metrics DeletionQV, IPD, InsertionQV, PulseWidth, QualityValue, MergeQV, SubstitutionQV, DeletionTag’. The polymerase kinetics information was loaded after alignment from the raw h5 format files. Finally, three post-aligned datasets were sorted using cmph5tools and 6mA modification was identified using ipdSummary.py script with parameters ‘—methylFraction—identify m6A—numWorkers 16’. The methylated sites with low depths were filtered by using the method reported in Xiao et al.’s study [50]. Due to the different sequencing data depth of the three samples, the depths of the identified 6mA sites that were less than 37, 30 and 18 coverage in autosome chromosomes and 18, 15 and 9 in sex chromosomes were filtered out in AK1, HX1 and HG00514, respectively (Figure S2). Methylated loci occurred on the positive coding gene strand, suggesting that these genes underwent 6mA modifications.

### 4.3. DNA and RNA Genetic Variation Samples

The short-read data from the same HX1 and AK1 samples were used to identify the DNA and RNA genetic variations (Table S1). To clarify whether the effect of DNA methylation on DNA and RNA variation was universal, we used HeLa cells from different samples to generate DNA and RNA short-read data and identify genetic variations. DNA sequencing data of the HeLa cells were downloaded from SRA accession numbers SRR10083957 and SRR8802185 in PRJNA529767 and RNA from SRR13333554 in PRJNA688745 (Table S1).

### 4.4. DNA and RNA Datasets for Genetic Variation Analysis

Raw short reads of DNA and RNA from HX1, AK1 and HeLa cells were treated and quality-controlled using FastQC v0.11.6 (https://www.bioinformatics.babraham.ac.uk/projects/fastqc/; accessed on 3 September 2018) and trimmed adapter [90]. The paired-end fastq files from DNA and RNA were aligned to the GRCh38 reference genome using BWA-MEM and Tophat (v2.1.1) software programs with default parameters, respectively [91,92,93]. Reads were sorted and marked as duplicates using the Picard tool (http://broadinstitute.github.io/picard; accessed on 3 September 2018) after generating an alignment BAM file. SplitNCigarReads was used to reformat the alignments to span introns for RNA-seq data with default parameters. Finally, we used GATK HaplotypeCaller, GenotypeGVCFs and ApplyBQSR (Base Quality Score Recalibration) to identify the genetic variations of DNA and RNA in the samples [94,95]. The variation identification and genotyping were performed across the GRCh38.p12 reference genome. Identified variants were filtered using VQSR with parameters ‘-an MQ -an QD -max-gaussians 8 -tranche 99.0′. DNA SNV was further screened by the consistency of dbSNP positions and RNA SNV was screened by depth >30X [96].

### 4.5. Statistical Analysis of Dynamic Transcriptional Genetic Variations

**Figure 5:**
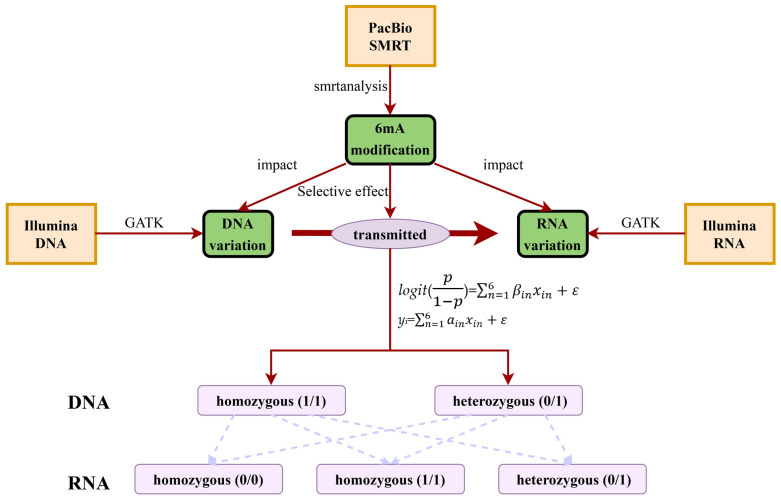
An experimental workflow.

For diploid alleles, the genotype of each variation was in three statuses: 0/0, 0/1 and 1/1, where 0 and 1 represented the normal and mutation, respectively. Therefore, the dynamic transcriptional genetic variation from DNA to RNA had six types: 0/1_0/0, 0/1_0/1, 0/1_1/1, 1/1_0/0, 1/1_0/1 and 1/1_1/1. To better reflect the relationship between DNA 6mA modification and transcriptional genetic variation, we divided them into two situations: i) comparison of the variation number in genes with and without methylation and ii) for methylated genes, the relationship between the methylation level (6mA density) and the number of genetic variations was statistically analyzed. For the first situation, all genes were divided into two groups using 6mA density. One group was Y = 0 if the genes had no methylation (6mA density equaled 0) and the other group was Y = 1 if the genes had methylation. The relationship between DNA 6mA methylation and transcriptional genetic variation was represented by the logistic regression model: *logit*($\frac{p}{1-p}$) = $\sum_{n=1}^{6}\beta_{in}x_{in}+\varepsilon$, where *p* is the probability of the event that Y = 1, *β_in_* and *x_in_* refer to the regression coefficient and variation type number of the *i*th gene and *ε* was the residual term. For the second situation, we constructed a linear model to analyze the relationship between 6mA density and the number of variations. The linear model was *y_i_* = $\sum_{n=1}^{6}a_{in}x_{in}+\varepsilon$, where *y_i_* was the 6mA density of the *i*th gene, *a_in_* and *x_in_* refer to the regression coefficient and variation type number of the *i*th gene and *ε* was the residual term. Based on the above models, the significant transcriptional genetic variation types associated with 6mA densities were selected and statistically analyzed (Figure 5). We used R 3.1.0 to perform the Pearson coefficients calculation, statistical analysis and draw figures in this study. The custom Perl script was used to process the text files. Different gene regions (exon, intron and UTRs) from gtf annotation file were obtained by bedtools and shell script. The function annotation of GO and KEGG was used with DAVID and the threshold value (*p*-value) was 0.01 [97,98].

### 4.6. 6mA Methylation in Imprinting Genes

Imprinting genes are a small subset of genes, in which one copy is turned off in a parent-of-origin-dependent manner and refers to an epigenetic marking of the genes [99,100]. To further validate the effect of DNA modification on dynamic transcriptional genetic variations, we collected 203 imprinting genes that were validated by reports and experiments from the geneimprint database (https://www.geneimprint.com; accessed on 3 September 2018). Based on the reference annotation file of GRCh38, we found the region of these imprinting genes in the genomes. We located 6mA modification sites and variations on these imprinting genes using the bedtools intersecting with parameters ‘-loj’ (Table S10). Considering the background of non-imprinted genes, all genes were divided into two groups (imprinting genes and non-imprinting genes) and each group was further divided into methylated and unmethylated genes in HX1 and AK1. To access the effect of imprinting genes, we compared the methylated imprinting and methylated non-imprinting genes and the unmethylated imprinting and unmethylated non-imprinting genes. Then, to access the effect of the methylated genes, we further compared the methylated and unmethylated genes in the imprinting genes and the methylated and unmethylated genes in the non-imprinting genes. The numbers of DNA variations and transmitted variations from the above comparisons were statistically tested using a *t*-test.

## 5. Conclusions

In this study, we investigated the role of DNA 6mA modification in human genetic variations. The variations of DNA and RNA in methylated genes were significantly lower than in unmethylated genes. DNA 6mA modification retained the transmitted mutated alleles in heterozygous (0/1 to 0/1) and homozygous (1/1 to 1/1) variations from DNA to RNA in 6mA modification genes. The 6mA modification regulated DNA-transcribing RNA, which supports the central dogma.

## Figures and Tables

**Table 1:** 6mA modification loci and mapping genes in three samples.

Sample	No. of Methyloci ^1^	No. of Methyloci ^1^ on Genes	Ratio of Methyloci ^1^ on Genes (%)	Strand-Methylated Genes ^2^
HX1	753,242	238,597	31.68	25,926
AK1	852,570	280,650	32.92	27,096
HG00514	827,068	257,444	31.13	22,339

^1^: Methyloci refer to methylation medication loci. ^2^: Methylated genes refer to genes with methyloci.

**Table 2:** Statistics of DNA variations in HX1 and AK1.

Sample	Total Variations No.	No. of Variations in Genes	No. of Genes with Variations	No. of Methylated Variations	No. of Methylated Genes with Variations	No. of Genes with Methylated Variations
HX1	2,593,902	1,522,416	35,232	2270	22,280	707
AK1	2,788,637	1,638,029	35,685	2308	23,293	809

**Table 3:** Transcriptional variations from DNA to RNA.

Type	East Asian Samples			HeLa
	HX1	AK1	Consistency	
Total RNA variations	21,346	80,139	18,519	29,007
No. of variations in genes	21,044	77,791	18,345	28,632
No. of genes with variations	6630	13,129	6139	8481
No. of transmitted variations	7631	28,787	4219	6370
No. of genes with transmitted variations	3786	9309	2587	3841

**Table 4:** Logistic regression of consistent methylation sites and variations in HX1 and AK1.

Transmit Type	HX1		AK1	
	Estimate	Pr (>|t|)	Estimate	Pr (>|t|)
(Intercept)	0.0684	0.280968	0.077022	0.22456
0/1_0/0	1.57906	0.144657	−0.007638	0.99214
0/1_0/1	0.11667	0.000448 ***	0.103532	0.00264 **
0/1_1/1	−0.63351	0.174365	−0.736716	0.10425
1/1_1/1	0.13076	3.89 × 10^−5^ ***	0.136978	6.28 × 10^−6^ ***

**: *p*-value < 0.01, ***: *p*-value < 0.001.

**Table 5:** Linear regression results of consistent methylation sites and variations in HX1 and AK1.

Transmit Type	HX1		AK1	
	Estimate	Pr (>|t|)	Estimate	Pr (>|t|)
(Intercept)	1.27 × 10^−4^	<2 × 10^−16^ ***	1.30 × 10^−4^	<2 × 10^−16^ ***
0/1_0/0	4.69 × 10^−4^	2.93 × 10^−7^ ***	−6.50 × 10^−5^	0.6425
0/1_0/1	4.03 × 10^−6^	0.25	−2.81 × 10^−7^	0.9396
0/1_1/1	3.07 × 10^−5^	0.553	−9.57 × 10^−5^	0.0598
1/1_1/1	2.51 × 10^−6^	0.46	5.44 × 10^−6^	0.0892

***: *p*-value < 0.001.

**Table 6:** Logistic regression model results of consistent methylation sites and different genotype variations in HeLa cells.

Transmit Type	Estimate	Pr (>|t|)
(Intercept)	−0.09068	0.2267
0/1_0/0	0.17215.	0.0855
0/1_0/1	0.21859	7.35 × 10^−6^ ***
0/1_1/1	0.01122	0.8107
1/1_0/0	0.28214	0.4466
1/1_0/1	0.10845	0.4791
1/1_1/1	0.08597	0.107

***: *p*-value < 0.001.

**Table 7:** Statistical results of DNA variations in imprinting and non-imprinting genes in HX1 and AK1.

Classification	HX1		AK1	
	Methylated- Non-Imprinted	Unmethylated- Non-Imprinted	Methylated- Non-Imprinted	Unmethylated- Non-Imprinted
Methylated- imprinted	0.01	3.91 × 10^−7^	4.28 × 10^−3^	1.11 × 10^−7^
Unmethylated- imprinted	2.81 × 10^−292^	0.22	2.05 × 10^−238^	0.04

**Table 8:** Statistical results of DNA variations in methylated and unmethylated genes in HX1 and AK1.

Classification	HX1		AK1	
	Unmethylated- Imprinted	Unmethylated- Non-Imprinted	Unmethylated- Imprinted	Unmethylated- Non-Imprinted
Methylated- imprinted	4.55 × 10^−7^	3.91 × 10^−7^	1.47 × 10^−7^	1.11 × 10^−7^
Methylated- non-imprinted	2.81 × 10^−292^	0.00	2.05 × 10^−292^	0.00

## Data Availability

Three East Asian samples (HX1, AK1, and HG00514) were collected from the NCBI SRA database PRJNA301527, PRJNA298944, and PRJEB12236. HeLa cells were downloaded from SRA accession numbers SRR10083957 and SRR8802185 in PRJNA529767, and RNA from SRR13333554 in PRJNA688745.

## Supplementary Materials

The supporting information can be downloaded at https://www.mdpi.com/article/10.3390/ijms251910400/s1.

## Abbreviations

**6mA**	DNA N6-methyladenosine
**5mC**	5-methylcytosine
**SMRT**	single-molecule, real-time sequencing
**IPD**	inter-pulse duration
***KRT5***	keratin 5
***IDH2***	isocitrate dehydrogenase (NADP(+)) 2
***SRSF2***	serine- and arginine-rich splicing factor 2
***GPR15***	G protein-coupled receptor 15
***IGF2***	insulin-like growth factor 2
***SNRPN***	small nuclear ribonucleoprotein polypeptide N
**LC-MS/MS**	liquid chromatography–tandem mass spectrometry
**N6AMT1**	N-6 adenine-specific DNA methyltransferase 1
**ALKBH1**	alkB homolog 1, histone H2A dioxygenase
**6mACE-seq**	6mA cross-linking exonuclease sequencing
**6mA-IP-Seq**	6mA immunoprecipitation sequencing
**SLE**	systemic lupus erythematosus
