# Supplementary material for: Selective Effect of DNA N6-Methyladenosine Modification on Transcriptional Genetic Variations in East Asian Samples
Source: Int J Mol Sci. 2024 Sep 27;25(19):10400. doi: 10.3390/ijms251910400 (PMC11477068; doi:10.3390/ijms251910400)
Supplement: Supplementary file 1 [file ijms-25-10400-s001.zip › ijms-3196654-Supplement-figure.pdf]

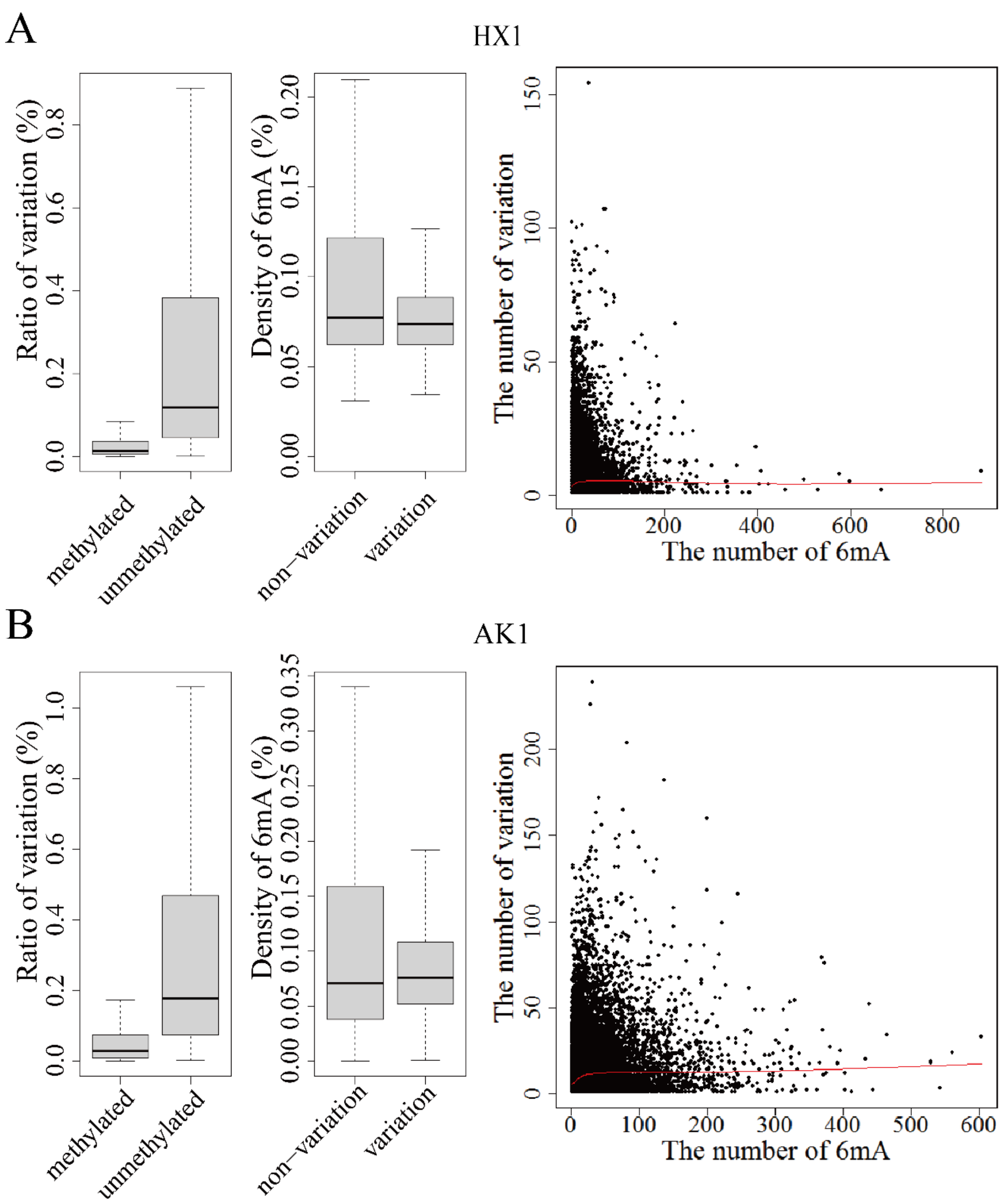

Figure S1. Compared 6mA density and ratio of RNA variations. (A: HX1; B: AK1.)

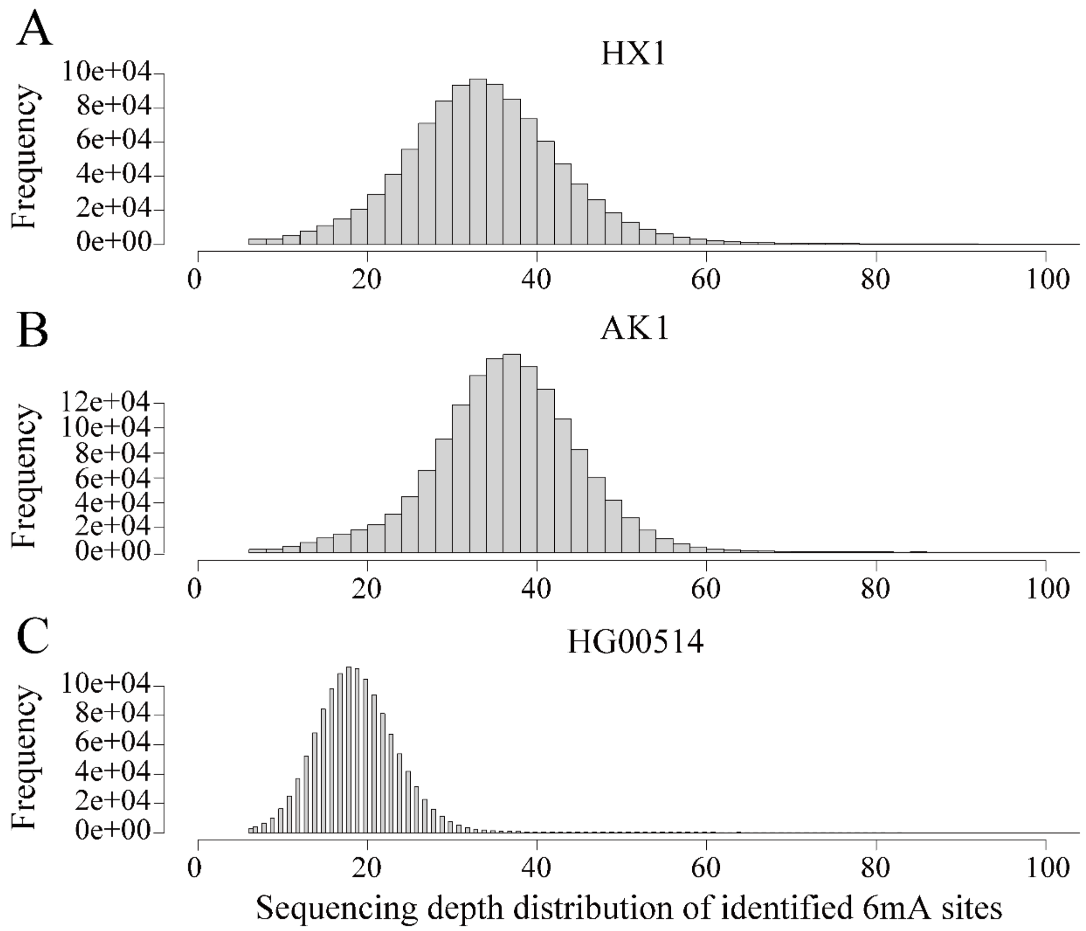

Figure S2. Sequencing depth distribution of 6mA modification in three human samples.

(A: HX1; B: AK1; C: HG00514)
